# Alterations in development of hippocampal and cortical memory mechanisms following very preterm birth

**DOI:** 10.1111/dmcn.13042

**Published:** 2016-03-29

**Authors:** Chiara Nosarti, Seán Froudist‐Walsh

**Affiliations:** ^1^ Department of Psychosis StudiesInstitute of Psychiatry, Psychology and NeuroscienceKing's CollegeLondonUK

## Abstract

Deficits in memory function have been described in children and adolescents who were born very preterm (VPT), which can have profound effects on their school achievement and everyday life. However, to date, little is known about the development of the neuroanatomical substrates of memory following VPT birth. Here we focus on episodic and working memory and highlight key recent functional and structural magnetic resonance imaging (MRI) studies that have advanced our understanding of the relationship between alterations seen in the VPT brain and typical neurodevelopment of networks supporting these memory functions. We contrast evidence from the episodic and working memory literatures and suggest that knowledge gained from these functional and neuroanatomical studies may point to specific time windows in which working memory interventions may be most effective.

## What this paper adds

- This article highlights how typical brain injuries associated with very preterm birth can interact with the typical development of episodic and working memory networks.
- By drawing on the lessons of 15 years of neuroimaging studies, suggested targets for cognitive interventions are proposed.

**Abbreviation**
VPT: Very preterm

Individuals who were born very preterm (VPT; <32wks’ gestation) are more likely to exhibit memory deficits compared with term‐born controls, which could impact their school performance and everyday life.^1^ In this paper we will focus on two different types of memory, episodic and working memory. Episodic memory could be described as the ability to encode, store, and retrieve information about an event, including information about the context in which the event took place. In contrast, working memory refers to short‐term storage and ‘online’ manipulation of information. Episodic and working memory are important components of executive control functions and are closely associated with each other. Working memory capacity has in fact been found to predict successful episodic remembering.^2^ These operations may interact via shared neuroanatomical mechanisms related to common control processes, as overlapping activation patterns – predominantly in prefrontal cortex – have been described for both episodic and working memory.^3^ However, distinct neural networks and activation patterns have also been proposed for episodic and working memory processing, and in this paper we will describe how these are organized in the typically developing brain. At a behavioural level, the capacity to form episodic memories is believed to emerge at around 4 to 5 years of age,^4^ and episodic memory performance improves during childhood until adolescence, with older children being more successful at integrating information with its contextual details.^5^ Such improvements may parallel the development of the brain's connectional architecture, which we describe in detail later in this review. Working memory abilities also emerge in early childhood and develop rapidly between the age of 4 years and 10 years. Much of the improvement in working memory capacity appears be closely associated with increases in processing speed, and with the development of subvocal rehearsal strategies.^6^

After summarizing the organization of episodic and working memory in the typically developing brain, we will examine the structural alterations and injuries that are likely to impact specific memory components in VPT samples. Thirdly, we will describe the mechanisms by which the brains of VPT individuals may attempt to overcome such structural alterations. Lastly, we will contrast the findings from episodic and working memory, and discuss the possible implications these may have for cognitive interventions that aim to ameliorate the cognitive profile of VPT children.

Research on the structural and functional memory correlates in VPT samples is presented systematically. Studies were identified using PubMed with the search terms (‘preterm OR VPT’ AND ‘MRI OR magnetic resonance imaging’ OR ‘fMRI OR functional magnetic resonance imaging’ AND ‘memory OR learning’). There were no restrictions on the year the studies took place. Studies in any language were considered. This search was supplemented by hand searching of reference lists of published articles. Studies were included if a group of individuals born preterm (including very or extremely preterm) were compared with a group of controls using quantitative structural MRI, and quantitative structural measures were correlated with memory outcome measures; or, for fMRI studies, if a group of individuals born preterm (including very or extremely preterm) were required to perform an fMRI memory task and activation was compared with a control group. The PubMed search produced 90 articles; 26 of these met the inclusion criteria. One additional article that was found through manually searching the references met the inclusion criteria. The outcomes of these searches were then divided into ‘structural’ (*n*=16) and (primarily) ‘functional’ (*n*=11) studies, and are shown in Tables 1 and 2 respectively.

**Table 1:** Structural magnetic resonance imaging (MRI) studies in relation to memory functions in very preterm (VPT) samples

References	Type	Cases (*n*)	Controls (*n*)	MRI modality	Memory measure	Mean age (y)	Results
Isaacs et al.^15^	ROI (hippocampus)	11 (<30wks)	8	T1‐weighted structural	RBMT; WMS form 1;CAVLT‐2; AMIPB‐Design Learning; RCFT; WISC‐III	13.5	VPT had lower scores on everyday memory tests. Minor differences in immediate, but not delayed recall. Reduced hippocampal volume in VPT. Hippocampal volume correlated positively with everyday memory ability
Caldú et al.^21^	ROI (corpus callosum)	25 (<33wks)	25	T1‐weighted structural	RBMT; RAVLT; RCFT;	13.5	Impaired everyday memory in VPT. Impaired verbal, but not visual memory. Corpus callosum area (at midline) correlated positively with everyday memory ability
Narberhaus et al.^22^	ROI (corpus callosum)	52 (<33wks)	52	T1‐weighted structural	RBMT;	14.2	Everyday memory deficit. Reduced area of genu, isthmus, and splenium of the corpus callosum. Area of genu and isthmus correlated with everyday memory ability
Kontis et al.^51^	Single tract (corpus callosum)	63 (<33wks)	45	Diffusion MRI	CVLT	19	Significant deficits in the majority of subtests (recall, recognition, long and short delay, perseverations). No significant FA or MD. Female VPT had greater MD than in female controls, particularly in the genu. There were no significant differences in MD between male VPT or control participants. In the preterm group, MD in the body of the corpus callosum correlated with the intrusions subscore. In the control group, MD in the genu and splenium was associated with the learning slope. MD in the body was associated with false positive responses
Fraello et al.^19^	Multiple ROI (cortical lobes and hippocampus)	49 (28±1.8wks)	20	T1‐weighted structural	WISC‐3; CTPP; CELF‐3	12	Deficit in short‐term recall of complex verbal sentences in VPT. No working memory deficits. No structure–function correlations in VPT group. In both groups combined, correlation between short‐term verbal memory and left temporal white matter volume
Skranes et al.^23^	ROI (entorhinal cortex thickness)	49 (bw<1500g)	58	T1‐weighted structural	Knox‐cube test (short‐term memory)	15	Impaired short‐term memory span in VPT, and reduced entorhinal cortex thickness. Left entorhinal cortex thickness was associated with short‐term memory ability (but not after controlling for socio‐economic status)
Allin et al.^26^	Whole white matter	80 (<33wks)	49	Diffusion MRI	CVLT; WMS	19	Verbal learning and global memory deficits in VPT, but not specific immediate and delayed recall deficits. FA reductions in corpus callosum, corticospinal tract, SLF 1, SLF3. FA increases in SLF3, external capsule. Corpus callosum, corticospinal tract, SLF 1, SLF3 associated with global memory in VPT
Thompson et al.^20^	Hippocampal shape and volume	184 (<30wks)	32	T1‐weighted structural	CVLT‐C‐long delay free recall; CMS‐long delay free recall dot locations.	Scans at term‐equivalent, neuropsychol. assess. at age 7	Visual and verbal memory deficits in VPT, and various hippocampal shape differences. No correlations between hippocampal shape and memory outcome. Hippocampal volumes at term (bilaterally) positively correlated with verbal memory outcome. Left hippocampal volume at term correlated with visual memory outcome, but not after correcting for intracranial volume
Brunnemann et al.^13^	ROI (hippocampal volume)	21 (<34wks)	19	T1‐weighted structural	AVLT (German version); RCFT	9	No episodic memory deficits in VPT, but bilateral hippocampal reductions. Trend between hippocampal volume and visual episodic memory in controls. No relationship in VPT (statistical comparison of correlation coefficients not reported)
Skranes et al.^42^	Whole cortex – surface area	38 (bw<1500g)	59	T1‐weighted structural	WAIS‐3 working memory subtest	19	Working memory deficits in VPT. Surface area reductions in orbitofrontal cortex, medial temporal lobe, insula, parietal lobe, occipital lobe, lateral temporal cortex. Correlation between working memory performance and cortical surface area in almost the entire cortex except for lateral parietal cortex
Omizzolo et al.^18^	ROI (hippocampal volume)	145 (<30wks)	34	T1‐weighted structural	WMTB‐C; CVLT‐C	7	Deficits on specific verbal working memory tasks and on specific short‐term memory tasks in VPT. Deficits in visual memory and learning. Deficits in short but not long delay verbal memory. Hippocampal volume reductions were present but did not survive correction for intracranial volume, gender, and neonatal brain abnormality. No relationship between hippocampal volume and memory performance
Thompson et al.^52^	Multiple ROI	96 (<30wks)	20	Diffusion MRI	CVLT – trials 1–5 part A	MRI, term‐ equivalent; neuropsych, 7	No verbal learning and memory deficits at age 7 in VPT. No FA differences. Greater mean (and axial and radial) diffusivity in VPT children. No relationship between diffusion indices and memory outcome
Molnár and Kéri^17^	ROI (hippocampus and caudate)	17 preterm infants with hypoxic injury	25, plus 14 with Fragile X syndrome	T1‐weighted structural	WMS‐R	22	This VPT group were matched on IQ to a group of individuals with Fragile X syndrome, and likely represent a lower‐performing VPT group. Deficits were present on all memory measures (verbal, visual, delayed, and general memory). Hippocampal volume reductions were present (also after correcting for intracranial volume). Fragile X syndrome patients had significantly larger hippocampi. VPT individuals and controls exhibited positive correlations between hippocampal size and general memory ability. Fragile X individuals exhibited the opposite pattern
Thompson et al.^12^	Longitudinal ROI (hippocampal shape and volume)	125 (<30wks)	25	T1‐weighted structural	WMTB‐C; CVLT; CMS (dot locations)	First scan, term‐equivalent; second scan and outcome, age 7	Deficits in verbal working memory and immediate spatial memory. VPT children exhibited less hippocampal growth and minor differences in shape development over the first 7 years. There were no correlations between hippocampal growth or shape development and memory outcome
Nosarti et al.^25^	Whole grey and white matter VBM	68 (<33wks gestation)	43	T1‐weighted structural	WMS‐R	20	Deficits in non‐verbal memory (immediate and delayed) in VPT. alterations: Grey matter: (VPT<Control) MTG/STG/striatum/thalamus/posterior vmPFC. (Control<VPT) anterior vmPFC. White matter: (VPT<Control) Bilateral temporal pole and fornix/thalamus/parahippocampal cortex (Control<VPT): Fusiform/lingual. Fornix/thalamus/parahippocampal cortex white matter volume was associated with non‐verbal memory score
Aanes et al.^16^	ROI (hippocampal volume)	44 (birthweight <1500g)	61	T1‐weighted structural	WMS‐3	19–20	Deficits in immediate auditory and visual memory in VPT. Deficit in delayed visual memory, but only a trend for delayed auditory memory. No deficit in delayed (auditory) recognition memory. Deficits in visuospatial working memory but not auditory (verbal) working memory. Bilateral hippocampal volume reductions (also when corrected for ICV) in VPT. Left hippocampal volume correlated with all immediate and delayed memory measures except for auditory recognition memory and did not correlate with working memory ability. In contrast right hippocampal volume correlated only with working memory ability

AVLT, Auditory Verbal Learning Test; AMIPB, Adult Memory and Information Processing Battery; CAVLT, Children's Auditory Verbal Learning Test; CELF‐3, Clinical Evaluation of Language Fundamentals – Third Edition; CMS, Children's Memory Scale; CTPP, Comprehensive Test of Phonological Processing; CVLT, California Verbal Learning Test; CVLT‐C, California Verbal Learning Test – Children's Version; FA, fractional anisotropy; ICV, intracranial volume; MD, mean diffusivity; MTG, middle temporal gyrus; R, revised; RAVLT, Rey Auditory Verbal Learning Test; RBMT, Rivermead Behavioural Memory Test; RCFT, Rey‐Osterrieth Complex Figure Test; ROI, region of interest; SLF 1, superior longitudinal fasciculus, section 1; SLF3, superior longitudinal fasciculus, section 3; STG, superior temporal gyrus; vmPFC, ventromedial prefrontal cortex; WAIS, Wechsler Adult Intelligence Scale; WISC‐3, Wechsler Intelligence Scale for Children – Third Edition; WMS, Wechsler Memory Scale; WMTB, Working Memory Test Battery for Children; WPPSI, Wechsler Preschool and Primary Scales of Intelligence.

**Table 2:** Functional magnetic resonance imaging (fMRI) studies in relation to memory outcomes in very preterm (VPT) samples

References	Type	Cases (*n*)	Controls (*n*)	Task	Age (y)	Results
Curtis et al.^53^	ROI (hippocampus and caudate)	9 (27–35wks)	9	Two tasks: a) DMS)/DNMS b) spatial memory span	13.8	No between group performance differences. No hippocampal activation differences on DMS/DNMS task. Spatial span task: no differences in right hippocampal activation; VPT showed greater activation in the right caudate nucleus during the encoding phase, but less activation of the same structure during the testing phase
Giménez et al.^27^	ROI (hippocampus and parahippocampal cortex) and uncorrected whole brain ROI of hippocampus also.	14 (<34wks)	14	Face‐name associative learning. Outside scanner: RAVLT (verbal), RCFT (visual).	14.7 (range 12–18)	VPT performed worse than controls during the task in both recall and recognition. Deficits in verbal learning (RAVLT) and a trend towards worse recognition memory (RAVLT). Deficit also present in visual memory (RCFT) ROI and VBM fMRI analysis: increased right hippocampal activation in the VPT group. Positive correlation between activation in the right hippocampus and recognition performance on online task for the VPT group only. Reduced hippocampal volumes bilaterally in VPT compared with controls, but only the left hippocampus was significantly smaller when covarying for intracranial volume. Positive correlation between right hippocampal volume and task activation
Narberhaus et al.^54^	Whole brain	21 (<33wks)	22	Visual paired associates	20	No between group performance differences. Encoding: (VPT>Control) Left caudate nucleus, right cuneus, left superior parietal lobule. (Control>VPT) Right inferior frontal gyrus. Recognition: (VPT>Controls) Right cerebellum, bilateral anterior cingulate
Lawrence et al.^28^	Whole brain ROI (hippocampus and parahippocampal)	22 (<33wks)	22	Verbal paired associates	20	No between group performance differences. Encoding (VPT>Control) Left parahippocampal gyrus, left precentral gyrus. Also right precentral gyrus, left posterior cerebellum (when contrasted with active baseline rather than resting baseline). Recall (VPT>Control) Left precentral gyrus. Grey matter reductions in bilateral hippocampus and increase in left parahippocampal gyrus in VPT. Positive correlation between left parahippocampal gyrus grey matter volume and task activation in VPT group alone
Taylor et al.^44^	Whole brain (unspecified level of correction)	10 (<32wks)	10	One‐back (short‐term memory component of the n‐back task).	7–9	No between group performance differences. VPT showed reduced activation in right parahippocampal cortex and precuneus
Kalpakidou et al.^29^	Whole brain+whole brain MRI	41 (12 with neonatal periventricular haemorrhage+ventricular dilatation [PVH+VD]; 17 with uncomplicated neonatal periventricular haemorrhage [UPVH]; 12 VPT with no neonatal periventricular haemorrhage [VPTN])	17	Verbal paired associates	20–24	No between group performance differences. Encoding: PVH+VD and UPVH activated the right middle frontal gyrus, which was mildly deactivated in the VPTN group and strongly deactivated in the controls. Recall: PVH+VD strongly activated the posterior cingulate, which was also activated, but to a smaller extent in the UPVH group, showed little response in the VPTN group and was deactivated in the controls. A linear trend analysis of the data revealed a linear trend in the grey matter volume PVH+VD<UPVH<VPTN<Control in the right superior temporal gyrus, right cerebellum, left middle temporal gyrus, right globus pallidus, and right medial frontal cortex. Structure–function relationships not significant
Griffiths et al.^45^	Whole brain (uncorrected) + ROI (anterior cingulate, uncorrected)	28 (<28 gestational wks or 1000lb birthweight)	28	Combined 2‐back (n‐back working memory) with stroop colour‐word (selective attention)	11	VPT had a greater reduction in accuracy when working memory load increased from 1‐back to 2‐back, with the effect slightly greater in the colour condition. Trend for longer reaction times in VPT. VPT showed less activation than controls, mainly in the occipital lobe, but also on the hardest (colour 2‐back) condition in the insula, supplementary motor area and lingual gyrus. The ROI analysis suggested less activation of the anterior cingulate in the VPT group, especially in colour conditions
Salvan et al.^24^	Whole brain (learning linear trend analysis)+diffusion tractography	21 (<33 gestational wks)	10	Verbal paired associates	20	No between group performance differences. Encoding: Controls showed a progressive decrease in activation in the anterior cingulate/caudate nucleus as the task repeated. VPT showed a progressive increase in activation in the same areas. Recall: Controls showed a progressive increase in activation in the hippocampus/parahippocampal cortex/thalamus across as they were asked to repeatedly recall learned paired associates. VPT showed a progressive decrease in activation in the same area. VPT exhibited decreased fractional anisotropy in white matter tracts connecting these memory‐related areas, including the fornix, the inferior longitudinal fasiculus, inferior fronto‐occipital fasciculus and the splenium of the corpus callosum
Murner‐Lavanchy et al.^46^	Whole brain and ROI	41 (<32wks gestational age)	36	Dot location visuospatial short‐term/working memory task+out of scanner shape location task.	7–12	No between group performance differences. Whole brain: reduced activation in VPT in posterior middle frontal gyrus/precentral gyrus ROI analysis: no activation differences in the middle frontal gyri, and increased activation in the superior frontal gyri in the VPT group
Brittain et al.^30^	Whole brain (learning linear trend analysis)	24 (<33wks gestation)	22	Visual paired associates	20	No between group performance differences. Encoding: Controls increased activation in the parahippocampal cortex, substantia nigra, left inferior frontal gyrus and right anterior cingulate, while VPT individuals decreased activation in these areas. Controls decreased activation in the cerebellum, right middle temporal gyrus and medial frontal cortex, while VPT individuals increased activation in these areas. Recognition: Controls progressively increased activation in the insula/claustrum/putamen, while VPT individuals reduced activation in this area. In contrast, VPT increased activation in the left cerebellum, which showed a progressive reduction in activation in the control group
Daamen et al.^47^	Whole brain	73 (<32wks gestation or <1500g birthweight)	73	One‐ and two‐back (from the n‐back working memory task).	26.5	No between group performance differences. Reaction times higher in the VPT group, but there was no task level x group interaction. VPT showed stronger load‐dependent deactivation of the right precuneus than controls. A similar pattern was found in the right cerebellum/parahippocampal gyrus in post‐hoc tests. Deactivation in the cerebellum/parahippocampal gyrus was associated with reaction time in the VPT group, but not controls (although the correlations were not statistically compared)

DMS, delayed matching to sample; DNMS, delayed non‐match to sample; PVH+VD, periventricular haemorrhage with ventricular dilatation; RAVLT, Rey Auditory Verbal Learning Test; ROI, region of interest; UPVH, uncomplicated periventricular haemorrhage; VBM, voxel‐based morphometry; VPTN, very preterm, no periventricular haemorrhage.

## Episodic memory

### Development of the episodic memory system

The hippocampus is a critical structure in the episodic memory system. It lies at the end of convergent processing streams of visual, auditory (from temporal visual and auditory areas, via entorhinal and perirhinal cortex), and spatial information (from parietal areas, including posterior cingulate, via parahippocampal cortex). The hippocampus is thus in an ideal position to bind object information (from temporal cortex) with spatial information (from parietal cortex), to form memories of complete episodes.^7^

**Figure 1:**
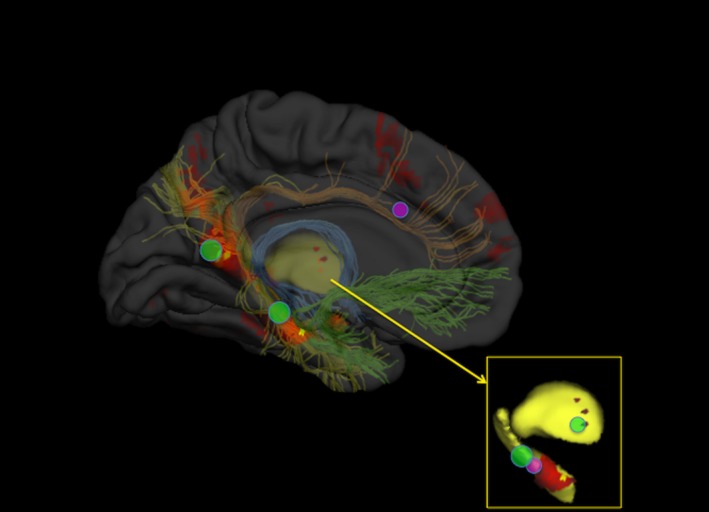
Functional alterations to the typical episodic memory network following very preterm (VPT) birth. Typical areas of activation during tasks of episodic memory are shown on the brain's left hemisphere (hot colours – activations are the result of an automatic meta‐analysis of 230 studies of episodic memory using neurosynth.org).^50^ White matter tracts that connect the hippocampus to other structures in the episodic memory system are shown beneath the cortical surface (fornix, blue; ventral cingulum, yellow; long cingulum, orange; uncinate fasciculus, green). The thalamus and hippocampus are shown in yellow in the main image and in the inset. Episodic memory structures that most commonly show reduced activation during functional magnetic resonance imaging (fMRI) tasks of episodic memory in VPT individuals compared with controls are marked with a green circle. Structures that show increased activation in VPT individuals compared with controls are marked with a pink circle. The size of the circle represents the number of fMRI studies that have reported over‐/under‐activation of the structure relative to controls.

The hippocampus develops rapidly during the first 2 years of life, and then more gradually until it reaches its peak volume between the ages of 9 years and 11 years.^8^ However, gains in episodic memory function continue after this point and are associated with increased specialization of hippocampal subdivisions, and with the structural development of hippocampal–cortical networks.^9^ This reflects the fact that while the hippocampus is the critical hub, it is just one part of a greater episodic memory system, which includes the mammillary bodies, anterior thalamic nuclei, posterior cingulate, and parahippocampal cortex. This functional loop is complemented by more direct hippocampal–cortical connections to medial temporal, posterior cingulate, and prefrontal areas, which are likely to be involved in the recall of information from the hippocampus (Fig. 1).^7^

The increased reliance on cortical support for episodic memory occurs in parallel with the maturation of the brain's connectional architecture. The fornix – the main hippocampal connection to other subcortical structures – reaches maturity early, at about 5 years of age.^10^ The cingulum and uncinate fasciculus, which connect the hippocampus to the posterior cingulate and prefrontal cortex, undergo a more protracted development,^10^ which is mirrored by increased fronto‐hippocampal functional connectivity and better performance on episodic memory tasks involving higher‐order functions.^9^

### Structural alterations to the episodic memory system following VPT birth

The periventricular location of the hippocampus makes it particularly vulnerable to damage following VPT birth. Reductions in volume in the hippocampus, as well as the interconnected thalamus and posterior cingulate cortex, are already present at term‐equivalent age and are related to the degree of prematurity.^11^ Smaller bilateral hippocampal volume in VPT samples compared with controls has been described at school‐age (~9% volume reduction),^12^, ^13^ adolescence (~14% volume reduction),^14^, ^15^ and adulthood^16^, ^17^ although some studies have found no hippocampal volume differences when correcting for factors such as intracranial volume.^18^, ^19^ Interestingly, hippocampal volume at term, but not at 7 years of age (or the amount of growth between these time‐points), has been associated with episodic memory abilities at age seven.^12^, ^18^, ^20^ This suggests that children with early hippocampal damage may have a limited capacity for the development of episodic memory functions. Structural and functional alterations in the fornix, corpus callosum, and the parahippocampal, entorhinal, and perirhinal cortices following VPT birth persist until adolescence^21^, ^22^ and early adulthood^23^, ^24^, ^25^, ^26^ and are correlated with memory ability.^25^ Table 1 summarizes studies investigating the structural brain correlates of memory functions in VPT samples.

### Intrinsic coping strategies and functional adaptation

As the brain structures centrally implicated in episodic memory do not appear to catch‐up developmentally, even by early adulthood, individuals born VPT may find alternative means of successfully completing tasks requiring episodic memory processing. Various studies have focused on how VPT individuals can bind information to form memories and later recall that information despite structural damage to the core circuit. In an early combined structural/functional MRI study Giménez et al. showed evidence that lateralized structural deficits to the hippocampus in a heterogeneous sample of VPT adolescents can be partially compensated by an increase in functional activation of the contralateral hippocampus.^27^ A similar study by Lawrence et al. found that while hippocampal volumetric reductions were present in the VPT group compared with controls, there was an increase in the volume of the parahippocampal cortex, with this increase being correlated with an increase in activation in this area.^28^ Kalpakidou et al. showed that increased levels of neonatal brain injury following VPT birth were associated with reduced access to key nodes of the episodic memory network, namely the posterior cingulate and lateral prefrontal cortex.^29^

In two recent studies, we analysed the dynamics of how VPT individuals and controls learn memory associations and showed that ‘pure’ memory deficits can be understood in the context of neuroanatomical alterations occurring during learning. Salvan et al. used a task in which participants were required to learn associations between two words across repeated experimental blocks, and were later asked to remember them during a cued recall task.^24^ By looking for patterns of adaptive activation we were able to detect learning signals in the brain, and crucially, see how these patterns differed between VPT and control participants. VPT individuals made increasing use of domain‐general cognitive regions (caudate and anterior cingulate cortex) during successive encoding trials, which was in sharp contrast to the typical habituation signal seen in control participants. While controls increased activation in the hippocampal–parahippocampal–thalamic section of the episodic memory circuit during recall trials, VPT participants reduced activation in these areas, possibly reflecting a search for alternative strategies due to suboptimal engagement of the core episodic memory network. This hypothesis was supported by the finding that such functional adaptations were linked to structural alterations in pathways linking these structures, including the fornix.^24^ A related study of the dynamic formation of visual memory associations by Brittain et al.^30^ revealed reduced recruitment of the hippocampus, parahippocampal, and posterior cingulate cortices in VPT adults during learning of visual paired associates. This study also shed light on how such memory tasks can affect other networks, with similar reductions in activation seen in dopaminergic regions, such as the substantia nigra, which is thought to enhance hippocampus‐dependent memory formations through reward–learning related mechanisms.^31^

Figure 1 shows a visual summary of functional alterations to the typical episodic memory network following VPT birth.

## Working memory

### Development of the working memory system

**Figure 2:**
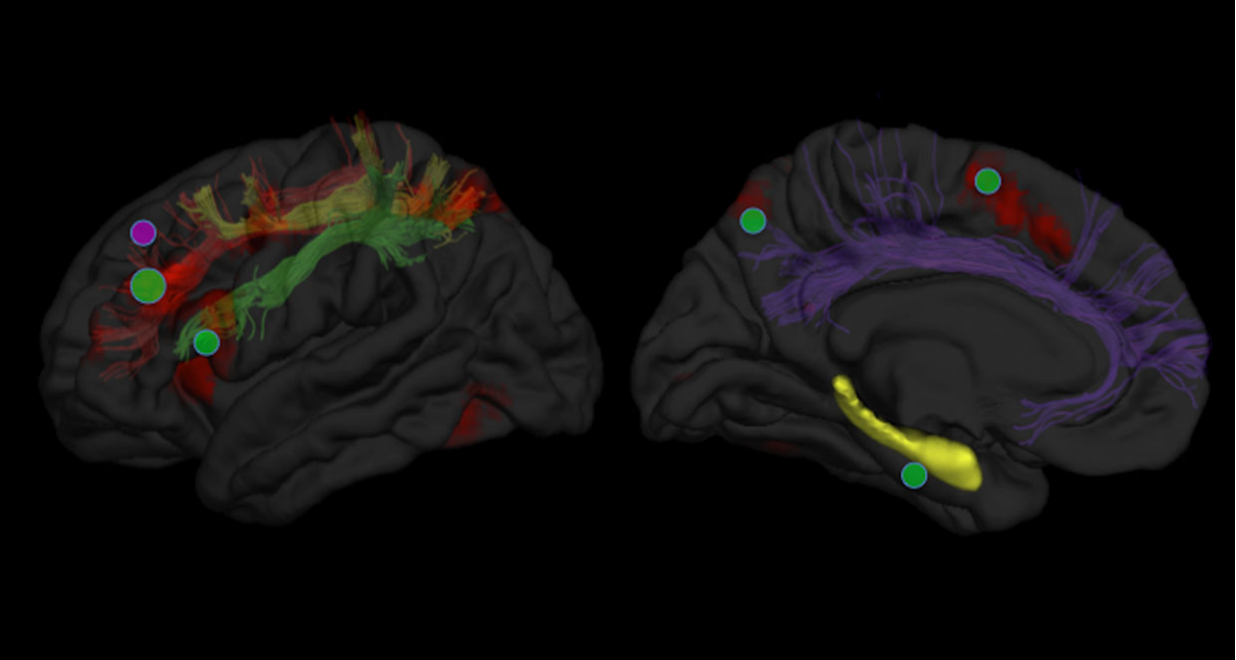
Functional alterations to the typical working memory network following very preterm (VPT) birth. Typical areas of activation during tasks of working memory are shown on the brain's left hemisphere (hot colours – activations are the result of an automatic meta‐analysis of 734 studies of working memory using neurosynth.org).^50^ White matter tracts that connect the fronto‐parietal structures of the working memory system shown beneath the cortical surface (SLF [superior longitudinal fasciculus]; SLF1, red; SLF2, yellow; SLF3, green; dorsal cingulum bundle, purple). The hippocampus is shown in yellow due to its apparent importance to childhood working memory deficits in VPT individuals. Working memory structures that most commonly show reduced activation during functional magnetic resonance imaging (fMRI) tasks of working memory in VPT individuals compared with controls are marked with a green circle. Structures that show increased activation in VPT individuals compared with controls are marked with a pink circle. The size of each circle represents the number of fMRI studies that have reported over‐/under‐activation of the structure relative to controls.

Current working memory capacity depends primarily on a fronto‐parietal system that involves dorsomedial, dorsolateral, and ventrolateral prefrontal cortex, as well as medial and lateral portions of the posterior parietal cortex.^32^ These regions are interconnected laterally by the three‐pronged superior longitudinal fasciculi and medially by the dorsal portion of the cingulum bundle (Fig. 2). Maturation of the working memory network is associated with functional activation increases in the frontal and parietal cortical areas^33^ and with cortical thickness decreases in prefrontal^34^ and posterior parietal cortex.^34^, ^35^ Concurrent micro‐ and macro‐structural development of the fronto‐parietal tracts is also seen and is associated with age‐related improvements in working memory capabilities.^35^ Furthermore, microstructure of the fronto‐striatal tracts and activation of the caudate nucleus during fMRI tasks have been shown to be predictive of future working memory capacity.^35^

The involvement of the hippocampus in working memory is strongly disputed, although it appears that dependence on this structure is seen mainly in childhood,^36^ possibly reflecting the comparatively early hippocampal development compared with the frontal and parietal areas that dominate the adult working memory system.

### Structural alterations to the working memory system following VPT birth

Working memory deficits have been described in childhood following VPT birth (see Anderson^37^ for a review). Prematurity‐related hippocampal damage at term‐equivalent age has been associated with working memory deficits at age 2 years.^38^ It remains to be seen whether these deficits are temporary and reflect the reliance of working memory function on the hippocampus that is seen early in development, or if early hippocampal damage disrupts subsequent neurodevelopment in brain areas sub‐serving working memory processing that can then result in permanent functional deficits. Interestingly, one recent study found a relationship between the volume of the right hippocampus and working memory ability in young adulthood,^16^ although to date this appears to be the only study to find such a specific link in adulthood. Furthermore, animal data suggest that early anatomical damage to the hippocampus may be sufficient to disrupt typical maturation of the prefrontal cortex and cause a permanent reduction in working memory abilities.^39^

In line with the possible role of striatal structures in the acquisition of working memory abilities in typical development described above, neonatal striatal and thalamic injury is predictive of working memory abilities at age 7 years in VPT children.^40^ In late adolescence, however, evidence suggests that working memory deficits following preterm birth are linked to the structural abnormalities of the mature working memory system observed in normative samples, such as cortical thickness alterations of the medial and superior parietal cortex^41^ and widespread cortical surface area alterations.^42^ Our recent work suggests that delays in fronto‐parietal cortical thickness maturation substantially diminish by early adulthood,^43^ although the functional correlates of such structural ‘catch‐up’ remain to be investigated in detail.

A summary of structural MRI studies in relation to memory functions in VPT samples is provided in Table 1.

### Intrinsic coping strategies and functional adaptation

Despite considerable neuropsychological evidence of working memory deficits in children born VPT, there have been surprisingly few fMRI studies investigating their functional neuroanatomical underpinnings. Taylor et al. used an fMRI task requiring basic visual working memory processing, and showed evidence of decreased activation in preterm children versus controls in regions belonging to both early and mature working memory systems, namely the right medial temporal and left medial parietal cortices respectively.^44^ These results suggest this phase of maturation (7–9y) may represent a key transitional stage of working memory development. Support for this argument comes from a study of 11‐year‐old children born extremely preterm by Griffiths et al., which shows activation reductions compared with controls in frontal working memory areas, which belong to mature (as opposed to early) working memory networks.^45^ Preliminary evidence of a catch‐up of activation patterns comes from a study by Murner‐Lavanchy et al.^46^ who showed that VPT children (7–12y) may have increased activation in superior frontal cortex to compensate for persistent functional deficits in the adjacent middle frontal gyrus.^46^ The most recent, and to date the largest, fMRI working memory study of VPT individuals found no working memory deficits in adulthood.^47^ Daamen et al., however, observed altered deactivations of the medial parietal cortex and a cerebellar node in their VPT group in the absence of ‘positive’ activation differences, which they hypothesized could be representative of compensatory adaptation. It is worth noting that the task used in the study by Daamen et al. was a relatively easy variant of the n‐back working memory task, and activation (or deactivation) patterns did not correlate with task performance. It remains to be seen what adaptations to the working memory system could lead to a catch‐up of function, and whether neonatal and environmental factors may modify activation and developmental patterns within VPT samples. We recently suggested that individuals born VPT with neonatal periventricular injuries may exhibit reduced prefrontal activation during working memory performance in adulthood compared with those without neonatal injury, despite having comparable activation on other tasks of executive function.^48^

Figure 2 shows a visual summary of functional alterations to the typical working memory network following VPT birth.

A summary of functional MRI studies in relation to memory functions in VPT samples is provided in Table 2.

## Conclusion

To date, studies of memory deficits in VPT samples have focused on episodic and working memory. The available evidence suggests that structural deficits to the hippocampus and connected structures following VPT birth may be long‐lasting, affecting episodic memory abilities from childhood to early adulthood. Functional and structural alterations to hippocampal–cortical networks are also associated with working memory deficits in VPT children. The lack of concluding evidence for an association between hippocampal‐related deficits and working memory function in adolescents or adults born VPT (with the exception of Aanes et al.'s study^16^) gives hope that the apparent transfer of working memory function from subcortical to fronto‐parietal cortex may result in neurodevelopmental adaptation. Functional neural compensation hypotheses are yet to be investigated in relation to memory processing, but promising results are provided by studies of other cognitive functions, such as language, which have suggested the existence of alternative task‐specific neural pathways in the developing preterm brain.^49^ Multimodal studies covering a greater range of ages and ultimately long‐term longitudinal studies need to be undertaken to provide a more comprehensive overview of the dynamics of development of memory function following VPT birth.

Although more research is needed, current evidence suggests that working memory may be a more promising target than episodic memory for cognitive training in VPT individuals. Moreover, interventions may be more effective if applied at an age when frontal and parietal cortices are sufficiently developed to be able to undertake such tasks, yet still maturing, so that focused interventions could exploit their residual neuroplastic capacity. Lastly, future research should include the identification of areas outside the typical memory‐specific networks that may be engaged in VPT samples as a result of neural adaptation, to successfully perform specific cognitive tasks.
